# Relations of the Dental Pulp to the Other Tooth Tissues

**Published:** 1878-10

**Authors:** L. C. Ingersoll

**Affiliations:** Keokuk, Iowa.


### ARTICLE II.

### *Relations of the Dental Pulp to the other Tooth Tissues.*

[CONTINUED.]

BY L. C. INGERSOLL, KEOKUK, IOWA.

Let us now examine the dental foramen to see if it does not teach us a lesson pointing to the same fact which I have been attempting to establish. We find that in childhood and in youth, during the period of most rapid development of tooth structure, the dental foramen is wide open, permitting the passage of many vascular canals, freighted with tooth material, for the building up process. But with the progress of the work this gateway gradually closes. The dental foramen becomes smaller and smaller, till at length we find it scarcely perceptible to the unaided eye. Here there is a reduction of the capacity for nutrition in proportion to the demand of the structure for nutrient support, and further evidence of the diminishing importance of the pulp.

I propose now to consider some objections to the view here presented.

The objection which most readily finds utterance is that to which I have alluded as a popular impression, and accepted, too, by many of the profession, viz: that the pulp is an absolute and essential factor, not only in the construction of the tooth, but in its preservation—that it holds its elements from dissolution. When popularly stated it is this: "When the nerve dies the tooth will rapidly decay." When stated by the profession it is this: "When the circulation of nutrient material has been cut off by destruction of the pulp, the dentine will surely tend to disintegration."

In answering the objection in the first form stated, I need only allude to the numerous cases of pulpless teeth standing in the mouth in all their integrity—save the decay that wasted them while the pulp was alive—and such teeth have stood for many years. A little inquiry will bring to our notice, almost daily, in the mouths of our patients, teeth still firm that have been pulplesss for ten, twenty or thirty years.

Should the pulp be devitalized in early life, all dentists would regard the chances of preservation of the remaining structure less favorable than when the devitalization occurs late in life, because of the greater porosity of the hard structure in early life. Yet the instances are numerous of boys and girls having teeth knocked loose by a blow or a fall, which perfectly destroyed the vitality of the pulps, and such teeth have been preserved, though greatly discolored, till advanced manhood or womanhood. Not long since I discovered two dark pulpless teeth in the mouth of one of my patients fifty-five years of age. On inquiry I learned that the accident occured at fourteen years of age by the kick of a colt. These teeth were firm and free from decay. It needs but little inquiry to learn of cases sufficiently numerous to satisfy the populor mind on this point.

But to answer the objection to the second form stated, coming from the professional standpoint. I am aware of the preconceived notions and theories which persistently assert themselves; gaining strength by repetition. An objection, representing the views of a large number of the profession on an important point in theory and practice is worthy of a candid consideration.

The objection is based upon the nutrient function of the pulp. It is a commonly received opinion that there is a perpetual change of tissue throughout the entire organism —that it requires but a few years at most to effect so complete a change that not one atom of old tissue remains. This is an old doctrine. It antedates dentistry and dental science. Its proofs were given to the world before the peculiar structure and organization of the teeth were known, or even before it was well understood that they had anything peculiar in their organization and structure—anything to distinguish them from bones. Has modern investigation ever given any proofs that the teeth are subject to the law of waste and supply in common with other tissues of the body? We know that the organizing functions treat the teeth in a very different manner from bones to which they are nearest allied. Bones may be wasted by disease, portions removed by fracture, or cut away by a surgical operation, and the lost parts will be restored by the organizing functions. Not so the teeth. When wasted by disease, abrasion, erosion, or a dental operation, no part of the lost tissue of a tooth is ever replaced. Dentists sometimes talk about nutrition of enamel. Why, then, is the enamel organ counted out, after the emergence of the teeth from the gum, and no longer numbered among tooth tissues? Where is the possibility of organic function being carried on with but one per cent. or three per cent. of animal tissue, while the ninety-seven or ninety-nine per cent. is of such crystalline density as to exhibit no more vascular trace than quartz? True, sensation has sometime been discovered in the enamel, and an occasional dentine fibril has turned up a loop into the enamel. But what can such exceptional cases prove more than the possibilities of abnormal structure?

Nutrition implies consumption and waste of tissue through the activity of animal functions. Where there is no waste of material there is no necessity for replacement. A structure that is composed chiefly of perishable material, like the soft tissues of the body, must of necessity require perpetual supply of new material, which we call nutrition, else the whole structure is soon broken down. In proportion as the structure is built up of *imperishable* material—not absolutely such, but imperishable as it stands related to existence of the individual—in like proportion is the necessity for replacement by nutrition diminished.

The teeth are composed so largely of imperishable material, that it is not in accordance with the philosophy of organic life to presume, in the absence of incontestible proofs, that the hard and imperishable material would be replaced simply because the soft and perishable animal tissue is continually undergoing replacement. This would be a waste of vitality and function, of which nature is never guilty. All observed facts point to the teeth as a peculiar structure, not subject, like other tissue, to the law of waste and supply, requiring nutritive functions of the pulp only to preserve intact their perishable animal tissue.

Again, if the matured tooth in its undiminished whole, need the nutritive function and vital support of the pulp to preserve it from disintegration, every portion of the tooth needs it alike. Nature pursues a damaging course and greatly blunders when she calcifies the dentine fibrils, and thus cuts off sensation and vascular action from any portion of the dentine, if such connection with the pulp is *necessary* to preserve the integrity of the dentine. All dental observation is a defense of nature—for we find the most thoroughly calcified teeth to resist decay the longest. These hard, dense teeth, that can be drilled into and excavated without pain, are the most enduring structures known to us; while the teeth that have large pulps and large dentine fibrils, fully active for nutrition, but not for calcification, are teeth readily disintegrated, and which give no promise of long continuance.

Let us look still farther at portions of the dentine cut off from pulp nutrition by decay, instead of by the physiological process of calcification. In every tooth penetrated by extensive decay, many of the dentine tubes are cut off, and, of course, the outlying dentine is cut off from all direct communication with the pulp. But this does not necessarily cut off all pulp nutrition; for the plexus formed near the border line of the dentine and enamel by the anastomosis of the dentine tubes and the looping of pulp fibrils is adequate to sustain vitality, for a while, in every part of the peripheral portion of dentine. But in numerous cases of extensive decay we find some portions of the walls of the cavity void of sensation, by reason of the death of the pulp fibrils. And we are all familiar with the fact, too, that when found very sensitive, if such cavities are filled and reopened after six months or a year, we often find the fibrils dead, although the body of the pulp be yet in a condition of activity and life; or the same non-sensitive condition of the walls of the cavity may have been brought about by calcification of the plexus. In either case, pulp nutrition is cut off from a portion of the dentine. And we are familiar with the further fact, that if such cavities are thoroughly filled, the whole tooth—including the devitalized portion—is preserved. Thousands upon thousands of such cases witness to the truth of this statement.

The conclusion is, that if part of a tooth in such condition may be preserved from disintegration, the whole tooth may, under favorable conditions, be preserved indefinitely from disintegration, though freed from pulp nutrition by the death of the pulp.

Another objection, or another form of the same objection, is found in the statement often made, that vitality protects the teeth from decay. Why, then, are the teeth not protected from decay? as they all have pulps. It is popularly said, too, that it is the special purpose of the enamel to preserve the teeth from decay. Why, then, with both these safeguards, are not the teeth protected from disintegration by caries? I take it that the teeth are enameled to protect them from abrasion—that they are enameled for the same reason that instruments made of iron are pointed with hardened steel, or case-hardened—to protect them from waste of substance by use, or to give them a cutting edge. Protection from decay is only an incidental and concomitant fact.

*Protection* from decay by vital action in the calcification of the dentine fibrils, is in perfect harmony with the views here presented. But *arrest* of decay through vital action is of rare occurrence. Some have seemed to convey the idea in their remarks on this subject, that the *mere presence* of vitality inherent in a living pulp had some mysterious power to prevent the action of those agencies which produce decay. Facts are strongly against this notion. I need in this connection but to again refer to the rapid decay of youths' teeth from the age of fourteen to twenty, when they are in the condition of the most active vitality. Another significant fact bearing on this point is, that recent decay is seldom found in the non-vital portions of the teeth of old people; but you look for fresh decay about the necks of the teeth where vitality is still active in dentine.

In suggesting any modification of practice based upon the positions which I have taken on the relations of the dental pulp to the other tooth tissues, I am aware that the conservatism of the world is such that mankind are struck with alarm when any note is sounded not in harmony with cherished theories and adopted practices. For a number of years past we have been lulled to rest, concerning exposed pulps, by the narcotism of creasote and oxychloride of zinc. The profession has been led to believe that the destruction of the pulp is well nigh equivalent to the destruction of the tooth; that therefore the slightest evidence of lingering vitality however feeble, is a sufficient warrant for persistent treatment ending with the oxychloride capping; and that such means are specific in restoring the healthful condition and functional activity of the pulp. Hope is a good thing—a most excellent quality of mind, and trust for a realization of the thing hoped for is a counterpart of the same. But hope and trust must have a basis of facts sufficiently broad and deep, else hope and trust are not reasonable.

I ventured the opinion, at the last meeting of the American Dental Association, that we had hoped in many cases without good foundation, and trusted too implicitly in the treatment, and believed too confidently in results. I hold these opinions still. I would not have the profession abate one whit the desire, to preserve exposed and diseased tooth pulps, nor the study and experiment requisite to that end. But I would have them diagnose more thoroughly their cases, observe more critically their successes, and modify their practice by a more careful judgment of physiological and pathological facts.

Dentists must acknowledge that the diagnosis of a diseased pulp is extremely difficult; not so much as to its present condition as to its vital power and susceptibility to treatment. If we bear in mind the facts as set forth in this paper, I think we shall conclude that the pulp has not a *tenacity of life*, except under the most favorable conditions. Its gradually diminishing substance and gradually diminishing functions, and its tendencies toward obliteration, should be considered; and should enter into a diagnostic opinion as to the results of treatment.

I believe that a judgment based upon sensation, or the impression of pain, has led many a hopeful and anxious dentist astray. It is not a very uncommon occurrence to find teeth that have wasted away—the entire crown, including the pulp—without causing any serious pain or discomfort. The treatment of teeth having this tendency might greatly deceive an operator who trusted chiefly to sensation in making a diagnosis. This quiet condition of an exposed pulp is a fearfully morbid condition. To cap and fill over such a pulp because it is quiet enough to tolerate the presence of foreign material, as it has tolerated contact of the atmosphere and fluids of the mouth, will not arrest the tendency to devitalization. "Failure" may be written on the record in less than six months. I would not even attempt treatment of pulps in this class of teeth.

Again, dentists are no doubt often deceived by the quieting influence of anodynes and narcotics. A patient comes into the office having a tooth that has often been the cause of severe pain. The dentist applies the remedy and almost instantly the pain ceases; and so long as it is narcotized it gives the patient rest. To assume, therefore, that this freedom from pain is evidence of health and restored function is a false assumption. It is no more evidence after five or ten days than the first day.

While penning this part of my paper, two cases were presented at my office by a lady from a neighboring city. For three weeks she had been annoyed, and at times greatly distressed, with pain on the right side of her face. She identified the second superior bicuspid as the source of it. On examination I found her teeth well cared for, both personally and dentally. The teeth in that locality had been filled in a most skillful manner, so far as I could judge from external appearance.

The offending bicuspid had in it a large gold filling, inserted about a year and a half previous, also another in the first molar. I instituted inquiry to learn the facts and circumstances attending the operation. I learned that both teeth had been treated for supposed exposure or sensitiveness of the pulp, and capped with oxychloride of zinc. My diagnosis of the case was that in the bicuspid the pulp was in a high state of inflammation, involving the peridental membrane—that in the molar the pulp was wholly devitalized, decomposing, and abcess imminent on the palatine root. I drilled into the crowns of both teeth, found the capping as represented, and the pulps according to diagnosis, except that the pulp in the molar had not become as thoroughly disorganized as I anticipated.

No doubt these teeth stood on the record of a good operator marked "success." All painful sensation was allayed during his treatment and filling, and for six months after. Suppose that the operator in these cases had considered the tendency of the pulp to obliteration, and the slight tenacity with which it holds its vitality when diseased, or disturbed, even by slight causes; he might have judged it better to extirpate the pulps of these teeth at *first*, than to run the risk he did; though with his confidence in the popular treatment he *might not* have considered it *any* risk.

Yet every dentist should consider the risk, in view of the large number of failures in the treatment. He should consider the many teeth in which the pulps have died when decay had penetrated the dentine but slightly, and the operator had never thought of any risk in filling.

How many of us have been surprised on examining such cases in one or two years after filling, to find the pulps dead or dying? He should consider the number of cases in which the death of the fibrils has caused disease or death of the pulp.

He should consider the physical idiosyncrasies of the patient, the history of other cases, if any, in the same mouth; and the complete or incomplete organization of the teeth.

Considering the points above enumerated, we may well conclude that of the cases needing treatment of any kind, as a means of preserving the pulp alive, only the most favorable presented, offer hope of *permanent* success—that a chronically congested pulp, a suppurating pulp, a tumefied pulp, a dalf-destroyed pulp, each and all are extremely doubtful of results—so doubtful that the chances of permanent preservation of such teeth are better by means of extirpation of the pulps than by any treatment at present adopted for their preservation.

The large number of cases of spontaneous death of the pulp *after treatment*, and the damaging effect of such death on the root membrane, should be a serious warning to every operator.

In the mouth of an adult individual, a healthy, vigorous root membrane is of far more importance than an emasculated pulp. To endanger the normal condition of this membrane is a more sure endangering of the tooth than anything that can possibly happen to the pulp which does not necessarily involve the root membrane. How many teeth with healthy, vigorous pulps, are annually lost by a wasting away of the root membrane alveolar processes! And dentists as yet are able to offer but temporary check, except in a few cases, to the progress of the disease. I would say, then, in all cases avoid a slow spontaneous death of the pulp, if you would avoid a chronic disease of the root membrane.

It may be replied to this that extirpation of the pulp endangers in like manner the root membrane. I am aware of the delicate nature of the operation of extirpation. It is an easy matter to devitalize a pulp, but to extirpate it, is another and wholly different operation. If an operator allows himself simply to devitalize, and not remove wholly the debris from the root canals, he may surely expect trouble in the root membrane. Delicate and difficult as this operation is, in the flattened roots, time and patience in the use of the requisite means will accomplish it, and leave the peridental membrane unharmed. This being the case, we may expect a long continuance of a pulpless tooth in the mouth.

Here we are confronted with the objection that, after the death of the pulp, the root membrane being compelled to perform vicariously the function of the pulp, induces hyperæmia, and at length its own destruction. This objection is a *petitio principii.* It assumes, without proof, the main point at issue. In premise and conclusion, it is false to fact. Vicarious performance of function is in this case anatomically impossible; and as I have shown, I think, in this essay, it is not physiogically required.

If the peridentium is left unharmed by the death of the pulp the performance of its own legitimate functions is alone adequate to sustain the tooth structure in all its oral relations. The hard tissue having gained a sufficient degree of density and hardness to maintain that structure by a law of mineralization independent of the vital functions of the pulp will certainly on the death of the pulp, make no unusual demand on the peridentium for vital support. It no doubt gives its measure of support to dentine of the roots, and also to the dentine of the whole tooth, so long as the nerve plexus remains intact throughout the whole structure. Performing thus *its now* legitimate functions, it is enough.

In concluding this essay it seems necessary to guard against misapprehension, thoughtless antagonism of ideas, and unwarrantable conclusions; for some gentlemen may rise and say, "if the teachings of this paper be correct, I see no need of the dental pulp; we may as well extirpate the pulps of all the teeth at once, and save mankind from all contingencies of dentalgia and loss of teeth." A foolish objection, indeed! I will, however accept its rational aspect so far as to say that the contingencies alluded to are neither sequences of *healthy* pulps in *sound teeth.* For a similar reason ignorant operators have removed sound and healthy teeth of the first set to encourage the development of the second set. Leave nature to eradicate temporary teeth, and to obliterate pulps in her own time and in her own way. The practical bearing of this paper regards only the treatment of *diseased and dying pulps.*

Another may say, "If teeth arrive at maturity in density and hardness in any specified number of years, then it may be said that the pulp is of no further use. In that case I see no reason why we should not abandon pulp treatment altogether, and extirpate every exposed pulp."

This objection rests on a misapprehension and a misstatement of the teaching of this paper. The conclusion drawn from the decreasing size, decreasing functional power and decreasing resources of the pulp, was that the tooth structure arrives at a period of development fully adequate to every performance of function before middle life; and that therefore the pulp is of little importance in the economy of nature, compared with its importance in earlier life. I do not say of *no* importance. In any case of exposure, with little or no disease of the pulp, it is worth while to attempt to save *this little* of pulp function whenever it can be done with a reasonable degree of certainty. But that there is a period when the hard structure has arrived at such a degree of maturity, that, in case of disease, the *comparative value* of the pulp is to be considered, will be readily conceded; and with chances of final restoration to healthy function are also to be well considered the natural tendencies to obliteration, and the consequent feeble hold the pulp has on life. The question of *abandoming* treatment, pertains *only* to the class of badly diseased pulps.

Another will say, "I prefer to give the treatment, *in every case*, and take the chances; if death of pulp and abscess follow, let it come. I will then cure the abscess." In the prognosis of a diseased and dying pulp, something else is more worthy to be considered than simple abscess. Abscess induced by a slow ebbing out of the life, and slow wasting of the substance of the pulp, is not of the benign character of an eight-days' abscess, although the latter may inflame and swell with ten-fold more pain and discomfort to the patient than the former. It is the chronically congested condition of the root membrane that attends a slowly-forming abscess, and that so often remains without cure, after the abscess itself is cured, that should excite the alarm of every operator who will willingly run the risk of abscess in the confidence he has in an easy cure.

The preservation of the teeth in their entirety, is conservative dentistry. But when, by inevitable disease, the vitality or functions of two or more kinds of tissues are endangered, it is the part of wisdom to sacrifice that which is of least importance.

The whole thought of this paper has a basis in the comparative value of the pulp at different periods in life, which I have attempted to establish as a physiological fact. Its tendency to gradual obliteration, and its ready death from very slight causes—as from the presence of a small filling in a cavity, that has penetrated not more than half through the dentine—have been brought to your notice to show with what feeble tenacity the pulp holds its vitality, and to show its very limited susceptibility to treatment when diseased. Any modification of practice which may grow out of the view of the subject here presented should be based more upon the greater probabilities of the continuance of teeth in the mouth till old age, by the careful and skillful extirpation of an emasculated pulp, than upon any treatment popularly resorted to for its preservation.—*Transactions of N. Y. Odont. Society in D. & O. Science Magazine.*

